# Three-Dimensional Analysis of Busulfan-Induced Spermatogenesis Disorder in Mice

**DOI:** 10.3389/fcell.2020.609278

**Published:** 2020-12-17

**Authors:** Hiroki Nakata, Taito Nakano, Shoichi Iseki, Atsushi Mizokami

**Affiliations:** ^1^Department of Histology and Cell Biology, Graduate School of Medical Sciences, Kanazawa University, Kanazawa, Japan; ^2^Department of Integrative Cancer Therapy and Urology, Graduate School of Medical Sciences, Kanazawa University, Kanazawa, Japan; ^3^Department of Clinical Engineering, Faculty of Health Sciences, Komatsu University, Komatsu, Japan

**Keywords:** spermatogenesis, seminiferous tubule, 3D reconstruction, testis, busulfan

## Abstract

We examined if the distribution of impaired or normal spermatogenesis differs along the length of seminiferous tubules in disorders of spermatogenesis. For this purpose, three-dimensional (3D) reconstruction of seminiferous tubules was performed in mice with experimental spermatogenesis disorder induced by intraperitoneal injection of busulfan, and the areas of impaired and normal spermatogenesis were analyzed microscopically. The volume of the testis and length of seminiferous tubules decreased, and the proportion of tubule areas with impaired spermatogenesis increased depending on the dose of busulfan. With the highest dose of busulfan, although the proportion of impaired spermatogenesis was similar among individual seminiferous tubules, it was slightly but significantly higher in shorter tubules and in tubule areas near branching points. The tubule areas with impaired and normal spermatogenesis consisted of many segments of varying lengths. With increasing doses of busulfan, the markedly impaired segments increased in length without changing in number, whereas normal segments, although reduced in number and length, remained even with the highest dose of busulfan. Individual remaining normal segments consisted of several different stages, among which stage I and XII were found at higher frequencies, and stage VI at a lower frequency than expected in normal seminiferous tubules. We also examined if the distribution of impaired or normal spermatogenesis differs among different 3D positions in the testis without considering the course of seminiferous tubules. Although the proportions of impaired spermatogenesis with the minimum dose of busulfan and normal spermatogenesis with the highest dose of busulfan greatly varied by location within a single testis, there were no 3D positions with these specific proportions common to different testes, suggesting that the factors influencing the severity of busulfan-induced spermatogenesis disorder are not fixed in location among individual mice.

## Introduction

Approximately 15% of couples trying to conceive are infertile, with a male factor involved in 50% of cases (Corona et al., 2019). The most severe form of male infertility is non-obstructive azoospermia (NOA), which accounts for 5% of infertile couples (Krausz, 2011; Tournaye et al., 2017; Pan et al., 2018). Spermatozoa in NOA can usually only be isolated from the testes, thus the most effective treatment is testicular sperm extraction. However, sperm retrieval in NOA is successful only in approximately 50% of cases due to partial and heterogeneous preserved focal spermatogenesis. Therefore, an accurate predictor for sperm retrieval is desired.

Many clinical factors, such as testicular volume, serum follicle-stimulating hormone, and serum inhibin B, have been investigated as an accurate predictor, but none were useful. Recently, the ultrasonographically measured size of seminiferous tubules was reported as a strong predictor (Nariyoshi et al., 2020). Identifying the focal areas containing mature sperm by ultrasonography mostly depends on the skill of the technician, and if the probable areas were known in advance, the success rate of the identification may increase. Therefore, clarification of the distribution of spermatogenesis in the testis with NOA is required.

It is generally believed that the sites of preserved spermatogenesis in the testis with NOA are multi-focal and distributed diffusely and homogenously rather than being patch-like and distributed heterogeneously in particular regions (Silber et al., 1997; Schwarzer et al., 2013). On the other hand, several groups reported a correlation between testicular blood supply and focal spermatogenesis in NOA (Har-Toov et al., 2004; Herwig et al., 2004). These studies were conducted in human therapy, but morphological analysis of spermatogenesis disorder in the whole human testis is practically difficult. Therefore, studies using animal models of the disorder are needed to confirm the three-dimensional (3D) distribution of impaired or normal spermatogenesis in the whole testis.

Our group recently reported the high-resolution 3D structure of all seminiferous tubules in animal testes using serial paraffin sections and high-performance 3D reconstruction software (Nakata et al., 2015a, 2017a, 2021; Nakata, 2019). Moreover, we found that the sites where spermatogenesis first occurs postnatally are distributed preferentially in the upper and medial areas of the testis close to the rete testis in mice (Nakata et al., 2017a). Our procedure was thought to be applicable to analyzing the distribution of spermatogenesis in the testis with spermatogenesis disorders. In the present study, we prepared mice with different degrees of spermatogenesis disorder by changing the doses of busulfan injection, which is known to cause extensive degeneration of seminiferous tubules (de Rooij and Kramer, 1970; Bucci and Meistrich, 1987). Then, the 3D-reconstructed seminiferous tubules were analyzed to examine if the impaired or normal spermatogenesis was distributed preferentially in particular sites in seminiferous tubules or in the testis.

## Materials and Methods

### Animals

The present animal study was approved by Kanazawa University (approval number: AP-173897) and conducted in accordance with the Guidelines for the Care and Use of Laboratory Animals of Kanazawa University. C57BL/6 strain male mice were purchased from Nippon SLC, Inc. (Hamamatsu, Japan), and reared under standard 12-h light/12-h dark laboratory conditions with free access to standard food and water.

### Busulfan Treatment

Busulfan (B2635; Sigma, St. Louis, MO, United States) was dissolved in DMSO and diluted in an equal volume of sterile distilled water to a final concentration of 0, 1, 2, or 3 mg/mL immediately before injection. Six-week-old male mice (body weight approximately 20 g) received a single intraperitoneal injection of 0, 10, 20, or 30 mg/kg of busulfan (IP-0, IP-10, IP-20, or IP30).

### Tissue Preparation and Periodic Acid-Schiff-Hematoxylin Staining

At 12 weeks after busulfan treatment, the mice were sacrificed by cervical dislocation. The testis and epididymis were dissected out en bloc, fixed in Bouin’s solution or 10% formalin neutral buffer solution overnight, dehydrated in a graded ethanol series, and embedded in paraffin. Using the Bouin’s-fixed paraffin blocks, serial 5-μm-thick sections with intervals of 40 or 50 μm were made by cutting the specimen longitudinally in parallel to the plane involving both the testis and epididymis using a microtome, and then mounted on glass slides. The sections were treated with periodic acid-Schiff-hematoxylin (PAS-H) to stain the basement membrane of seminiferous tubules, as previously described (Nakata and Iseki, 2019). The sections were digitized using a whole-slide scanner (Nanozoomer 2.0-HT; Hamamatsu Photonics, Hamamatsu, Japan) with a 20-fold objective lens, and the resulting digital images of the sections were visualized with viewer software (NDP.view2; Hamamatsu Photonics).

### Immunohistochemistry

Using the formalin-fixed paraffin sections, immunohistochemistry (IHC) was performed as described previously (Nakata et al., 2015b, 2017b). The sections were incubated with antibodies against ZBTB16 (1:200 dilution; HPA001499, Sigma-Aldrich, St. Louis, MO, United States), GATA4 (1:50; sc-1237, Santa Cruz Biotechnology, Dallas, TX, United States), or SYCP3 (1:400; ab15093, Abcam, Cambridge, United Kingdom) followed by secondary antibodies labeled with Alexa Fluor 488 or 594 (1:400, Molecular Probes, Eugene, OR, United States), or with Alexa Fluor 488-conjugated lectin PNA (1:400, Molecular Probes). All sections were counterstained with DAPI (300 nM; Molecular Probes), mounted on glass slides with Fluoromount (Diagnostic BioSystems, Pleasanton, CA, United States), and examined with a fluorescence microscope (BX51; Olympus, Tokyo, Japan). Fluoromount was then removed and the sections were washed and stained with hematoxylin and eosin (HE).

### Reconstruction Processing

The 3D reconstruction was performed as previously described (Nakata et al., 2017a) with slight modification. Briefly, extraction of the PAS-H-stained basement membrane in digital images was performed using ImageJ software (NIH; Bethesda, MD, United States^1^) and Adobe Photoshop 2020 software (Adobe Systems, Inc., Mountain View, CA, United States). After extraction, the images were converted into gray scale in JPEG format with Adobe Photoshop 2020 software at a resolution of 1,816 nm⋅pixel^–1^. Using Amira 6.3.0 software (Visage Imaging GmbH, Berlin, Germany), the serial images were aligned automatically followed by manual adjustment, and the inside of the outlines of a selected tubule was colored in using threshold processing and traced from section to section. This procedure was repeatedly applied to all seminiferous tubules with different colors and they were then 3D reconstructed. To draw the core lines of seminiferous tubules, individual traced tubules in cross-sections were shrunk concentrically by 16 pixels in all directions, their resolution was changed to 29,056 nm⋅pixel^–1^, and they were then reconstructed into thin tubules, in which the core lines were drawn using the same software. The whole testis was also 3D reconstructed by filling the inside of their outlines by threshold processing. The position of the rete testis was defined as the mean coordinate of the connections of all reconstructed seminiferous tubules with the rete testis and shown with a black sphere.

### Assessment of the Degree of Spermatogenesis Disorder

Spermatogenesis is routinely divided into 12 stages in mice based on changes in the morphology of the acrosome and nucleus (Oakberg, 1956a; Hess and Franca, 2008; Ahmed and de Rooij, 2009; Meistrich and Hess, 2013; Nakata et al., 2015b; Nakata, 2019). The degree of spermatogenesis disorder in each section of seminiferous tubules in busulfan-treated mice was assessed by histological observations of cell associations using NDP.view2., and the tubule portions were classified into three types according to the degrees of spermatogenesis disorder: normal, in which all germ cells that define one or more stages are arranged; abnormal with spermatocytes, in which all germ cells that define any one stage are not arranged, but spermatocytes are present; and abnormal without spermatocytes, in which only Sertoli cells are present in most cases, but spermatids are occasionally present. These three types represented normal, moderately impaired, and markedly impaired spermatogenesis, respectively.

### Statistical Analysis

Data are presented as the mean ± standard deviation (SD). Comparisons among multiple data were performed with one-way or one-way repeated measures analysis of variance (ANOVA) followed by Bonferroni’s or Dunnet’s *post hoc* test, respectively, and differences with a *P*-value of less than 0.05 were considered to be significant. Pearson’s correlation coefficient test was performed to assess the magnitude and direction of the proportionality between two parameters, and a positive or negative relationship was acknowledged when the absolute *r*-value was greater than 0.2.

## Results

### 3D Reconstruction of All Seminiferous Tubules

**FIGURE 1:**
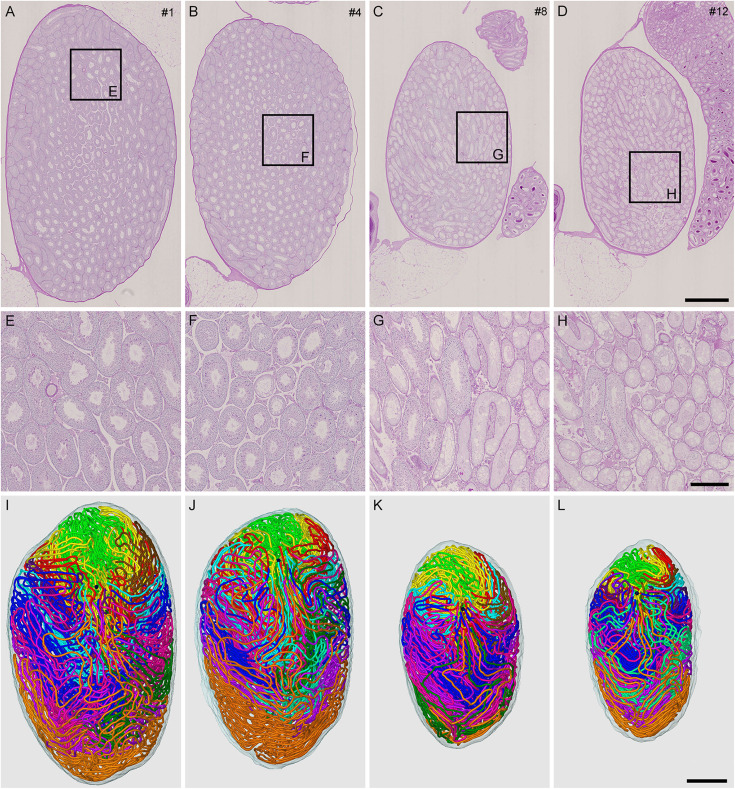
Reconstruction of all seminiferous tubules in mice treated with different busulfan doses (IP-0, 10, 20, and 30 for intraperitoneal injections of 0, 10, 20, and 30 mg/kg, respectively). PAS-H-stained longitudinal sections of testes from mouse #1 **(A,E)**, 4 **(B,F)**, 8 **(C,G)**, and 12 **(D,H)**, which represent IP-0, 10, 20, and 30, respectively, are shown at low **(A–D)** and high magnification (**E–H**, the framed areas in **A–D**). The 3D core lines of all reconstructed seminiferous tubules marked with different colors are shown (**I–L** for mouse #1, 4, 8, and 12, respectively) with the position of the rete testis (black sphere). Scales, 1 mm (**A–D** and **I–L**), 250 μm **(E–H)**.

To generate mice with different degrees of spermatogenesis disorder, 12 mice were divided into four groups of three mice each: IP-0 (#1, 2, 3), IP-10 (#4, 5, 6), IP-20 (#7, 8, 9), and IP-30 (#10, 11, 12), which received a single intraperitoneal injection of 0, 10, 20, and 30 mg/kg of busulfan, respectively. All seminiferous tubules were reconstructed in 12 testes, one from each mouse, at 12 weeks after busulfan treatment. Results in four representative testes, one from each group, are shown in Figure 1. As expected, the testis became smaller and seminiferous tubules appeared degenerated with higher doses of busulfan. The quantitative data of the testis and seminiferous tubules are shown in Table 1. None of the values for the numbers of seminiferous tubules, terminating points, and branching points per testis were significantly different among the four groups (*P* = 0.56, 0.92, and 0.98, respectively). On the other hand, the testis volume and total length of seminiferous tubules per testis significantly decreased in IP-20 and IP-30 (*P* < 0.05 between IP-0 and IP-20 or IP-30).

**TABLE 1:** Summary of reconstructed seminiferous tubules in busulfan-induced spermatogenesis disorder in mouse testes.

Testis	IP*	Number				Testis volume (mm^3^)		Total tubule length (mm)	
		Total tubules	Terminating points**	Branching points	Blind ends		Group average***		Group average***
#1	0	11	36	16	0	63.2	55.8 ± 5.4	1,815	1,701 ± 82
#2	0	10	38	18	0	50.4		1,629	
#3	0	13	42	16	0	53.8		1,659	
#4	10	10	36	16	0	55.6	49.2 ± 4.8	1,704	1,630 ± 67
#5	10	13	47	24	3	47.9		1,646	
#6	10	14	39	11	0	44.2		1,541	
#7	20	11	41	19	0	29.0	25.3 ± 2.8^†^	1,314	1,174 ± 103^†^
#8	20	11	36	14	0	24.7		1,138	
#9	20	13	43	19	0	22.4		1,071	
#10	30	12	41	21	0	24.0	22.1 ± 2.0^†^	1,074	1,049 ± 17^†^
#11	30	13	39	14	1	22.8		1,034	
#12	30	14	41	13	0	19.3		1,040	

*Intraperitoneal injection of busulfan (mg/kg); **Connections with the rete testis; ***Mean ± SD; ^†^Significantly different from IP-0.

### Analysis of Spermatogenesis Disorder in Seminiferous Tubules

**FIGURE 2:**
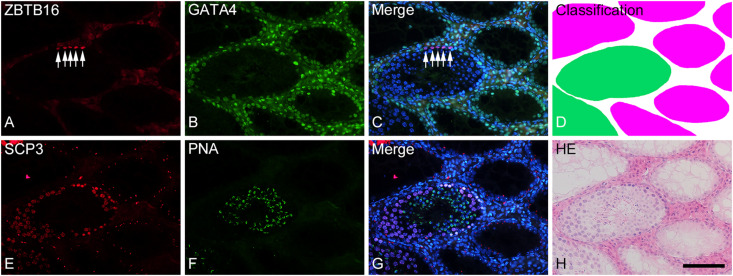
Photographs of a representative cross-section **(A–D)** and the adjacent cross-section **(E–H)** of a formalin-fixed IP-30 testis shown with double-fluorescence staining for ZBTB16 [**(A)**; red] and GATA4 [**(B)**; green] and for SCP3 [**(E)**; red] and PNA-lectin [**(F)**; green], respectively. **(C,G)** are merged pictures of **(A,B)** and **(E,F)**, respectively, together with nuclear staining with DAPI (blue). **(D)** represents classification of tubule areas with normal (green) and markedly impaired (red) spermatogenesis in the same section as **(A–C)**. **(H)** represents HE staining of the same section as **(E–G)**. In the normal tubules, type A spermatogonia (arrows) are stained in nuclei for ZBTB16 **(A)**, Sertoli cells are stained in nuclei for GATA4 **(B)**, spermatocytes are stained in nuclei for SCP3 **(E)**, and spermatids are stained in acrosomes for PNA **(F)**. In contrast, in the markedly impaired tubules, only Sertoli cells but no other cell types are stained with the respective markers. Scale, 100 μm.

**FIGURE 3:**
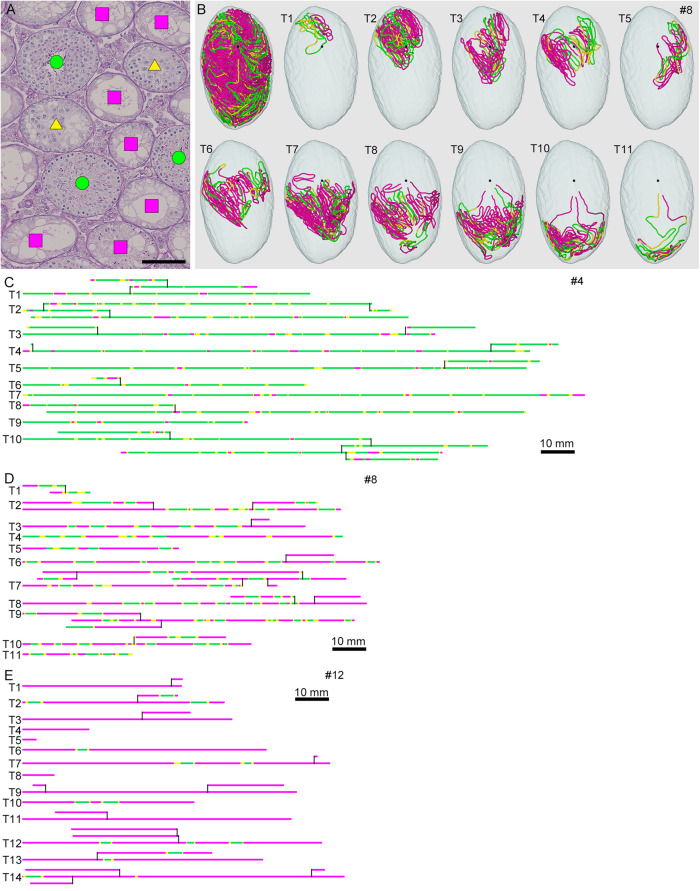
Classification of the degrees of spermatogenesis disorder in mice treated with different busulfan doses. All seminiferous tubules in each section are divided into three types: normal tubules (green circle), abnormal tubules with spermatocytes (yellow triangle), and abnormal tubules without spermatocytes (red square) [**(A)** for mouse #8]. The core lines of all reconstructed seminiferous tubules in #8, with areas belonging to the above three types marked with the corresponding colors, are shown in a vertical view with the position of the rete testis (black sphere) **(B)**. The scheme of all individual seminiferous tubules in mouse #4 (IP-10), #8 (IP-20), and #12 (IP-30) is shown in **(C–E)**. The segments composed of tubule areas belonging to the three types are marked with the corresponding colors. The branching points are shown with vertical bars. All free extremities of the tubules in these cases are connected to the rete testis in terminal points without forming blind ends. Scales, 100 μm **(A)**, 10 mm **(C–E)**.

**FIGURE 4:**
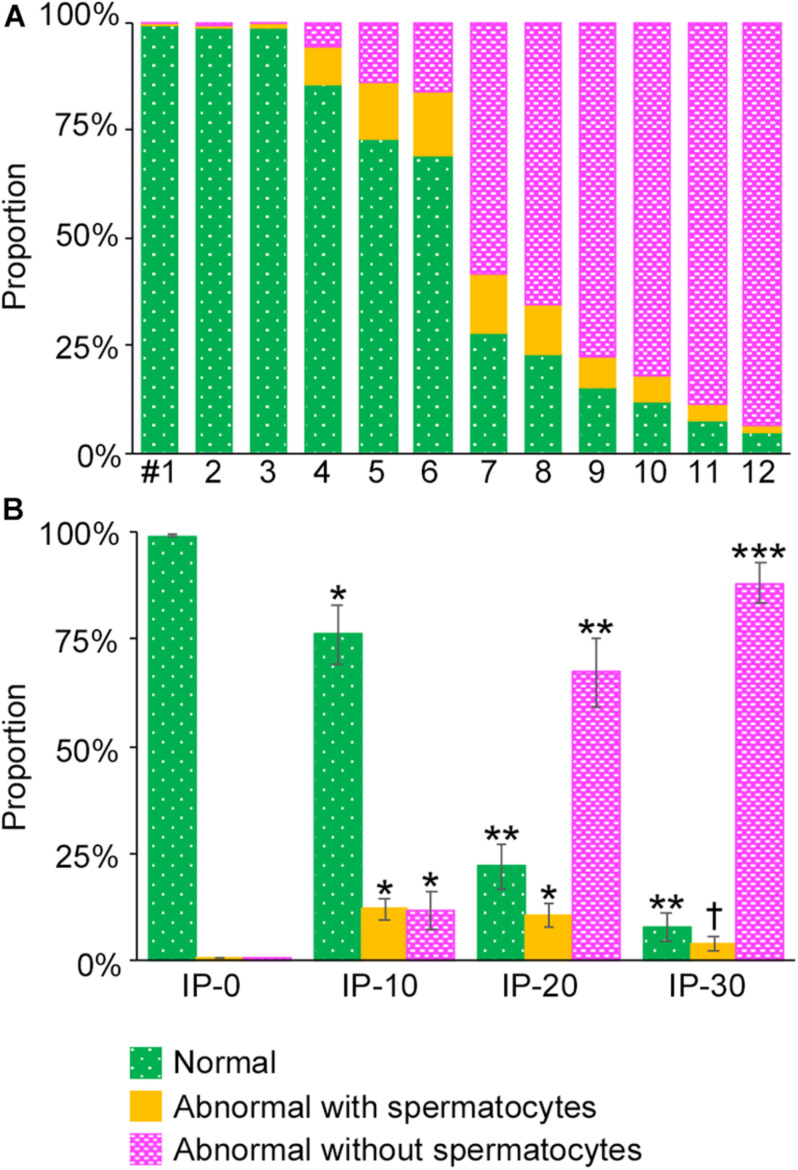
Proportions of different degrees of spermatogenesis disorder in the whole seminiferous tubules in mice treated with different busulfan doses. The proportions of tubule areas belonging to the three types marked with the corresponding colors are shown in individual mice **(A)** and in groups of mice (IP-0, 10, 20, and 30) **(B)**. Each value represents the mean ± SD of three samples. Statistical analysis was performed in **(B)** between values for the same colors. *Significantly different from IP-0 (*P* < 0.05). **Significantly different from IP-0 and IP-10 (*P* < 0.05). ***Significantly different from IP-0, IP-10, and IP-20 (*P* < 0.05). ^†^Significantly different from IP-10 and IP-20 (*P* < 0.05), but not from IP-0.

To analyze the degree of spermatogenesis disorder, all seminiferous tubule areas observed in each testis section were classified into three types as described in section “Materials and Methods,” i.e., normal, abnormal with spermatocytes, and abnormal without spermatocytes, which were represented by green, yellow, and red, respectively. Moreover, the normal tubule areas were confirmed to undergo a complete spermatogenesis by immunofluorescence staining for specific markers of type A spermatogonia, spermatocytes, and spermatids (Figure 2). Thus, the three colors represented tubule areas with normal, moderately impaired, and markedly impaired spermatogenesis, respectively (Figure 3A). According to this typing, the core lines of all seminiferous tubules in 12 testes were divided into three different color segments (Figures 3B–E). As the busulfan concentration increased, the proportion of normal segments (green) decreased (*P* < 0.05 between IP-0 and IP-10; between IP-10 and IP20 or IP30) and that of abnormal segments without spermatocytes (red) increased (*P* < 0.05 between IP-0 and IP-10; between IP-10 and IP-20; between IP-20 and IP-30) (Figure 4). On the other hand, the proportion of abnormal segments with spermatocytes (yellow) in IP-10 or IP-20 increased in comparison with that in IP-0, but that in IP-30 remained the same as in IP-0 and decreased in comparison with that in IP-10 or IP-20 (*P* < 0.05 between IP-0 and IP-10 or IP-20; between IP-30 and IP-10 or IP-20).

**FIGURE 5:**
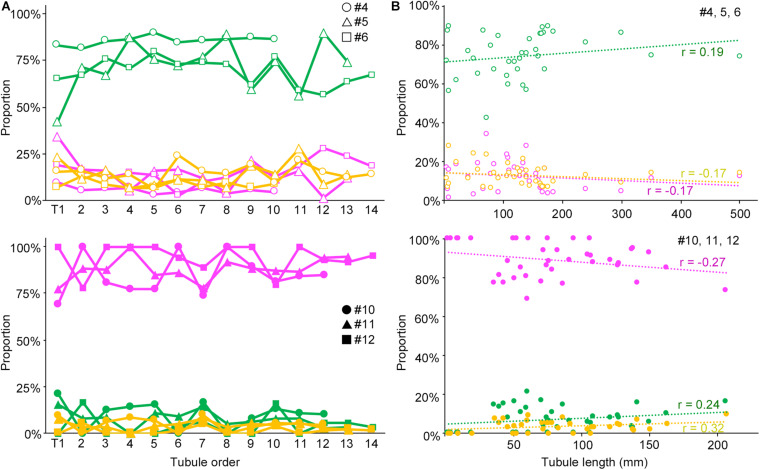
Relationships of the proportions of different degrees of spermatogenesis disorder with the order of all seminiferous tubules expressed as a line graph **(A)** and with the length of individual seminiferous tubules expressed as a scatterplot **(B)**. The upper figures are for IP-10 (#4, 5, and 6) and the lower figures for IP-30 (#10, 11, and 12). The colors represent normal tubules (green), abnormal tubules with spermatocytes (yellow), and abnormal tubules without spermatocytes (red). In **(A)**, the tubules are numbered from T1 to T10 (#4), to T12 (#10), to T13 (#5, 11), and to T14 (#6, 12) in the order of their connection with the rete testis from the top toward the bottom in the testis. The values are expressed with hollow circles, hollow triangles, and hollow squares for #4, 5, and 6, respectively, and with filled circles, filled triangles, and filled squares for #10, 11, and 12, respectively. In **(B)**, the values are expressed as hollow circles for #4, 5, and 6, and with filled circles for #10, 11, and 12. The *r* values of the regression lines are indicated.

We next examined if the degree of spermatogenesis disorder differs among different seminiferous tubules in a testis that were numbered in the order of their connection with the rete testis from the top toward the bottom, or among different seminiferous tubules of varying lengths in IP-10 and IP-30 (three mice each) (Figure 5). There was no notable relationship between the proportions of the three color segments and the tubule orders in IP-10 and IP-30. On the other hand, between the proportions of the three color segments and the lengths of individual seminiferous tubules, no correlation existed in IP-10 (|*r*| < 0.2), but weak positive correlations (*r* = 0.24 and 0.32 for green and yellow, respectively) and a weak negative correlation (*r* = −0.27 for red) existed in IP-30.

**FIGURE 6:**
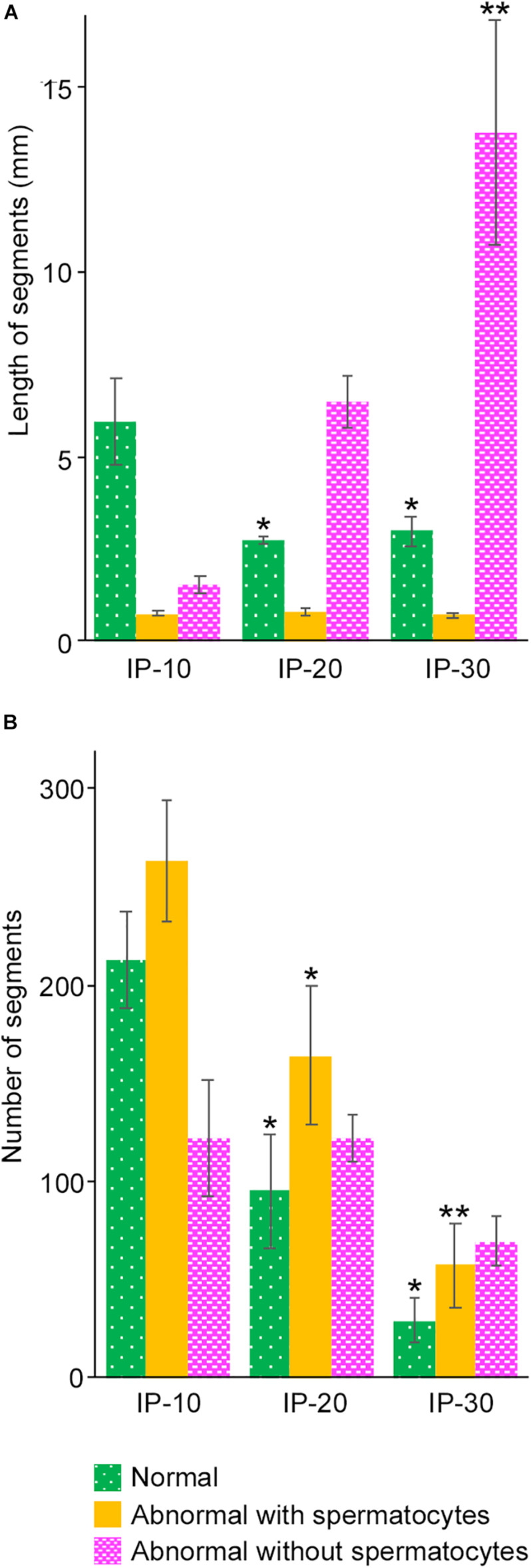
Mean lengths **(A)** and numbers **(B)** of the segments of seminiferous tubules with different degrees of spermatogenesis disorder in mice treated with different busulfan doses (IP-10, -20, and -30). The colors represent normal tubules (green), abnormal tubules with spermatocytes (yellow), and abnormal tubules without spermatocytes (red). Each value represents the mean ± SD of three samples. Statistical analysis was performed between values for the same colors. *Significantly different from IP-10 (*P* < 0.05). **Significantly different from IP-10 and IP-20 (*P* < 0.05).

As shown in Figures 3C–E, the areas of seminiferous tubules with different degrees of spermatogenesis disorder were distributed as many segments of varying lengths. We thus examined the changes in the length and number of these segments according to the different doses of busulfan. The total lengths and numbers of green, yellow, and red segments were measured in IP-10, IP-20, and IP-30 (Figure 6). The length of the green segments was significantly shorter in IP-20 than in IP-10 (*P* < 0.05), but remained unchanged in IP-30 compared with that in IP-20. The length of the red segments slightly increased in IP-20 compared with that in IP-10, although not significantly, and increased further significantly in IP-30 (*P* < 0.05). In contrast, the length of the yellow segments remained unchanged from IP-10 to IP-30 (*P* = 0.80). The number of green segments decreased significantly in IP-20 compared with that in IP-10 (*P* < 0.05) and slightly decreased further in IP-30, although not significantly. The number of red segments remained unchanged from IP-10 to IP-30 (*P* = 0.06). In contrast, the number of yellow segments significantly decreased in IP-20 compared with that in IP-10 and decreased further in IP-30 (*P* < 0.05).

**FIGURE 7:**
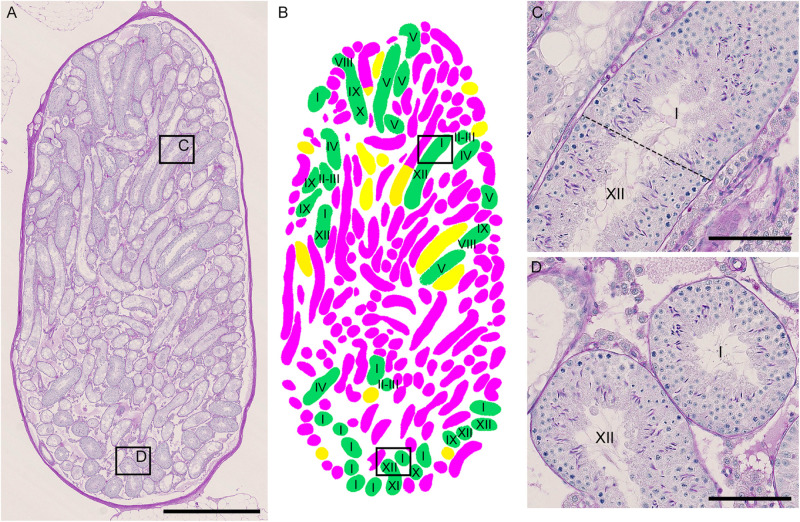
Photographs of a representative cross-section of IP-30 testis from #10 mouse shown with PAS-H staining **(A)** and with assessment of the degree of spermatogenesis disorder and identification of stages in individual tubule sections **(B)**. The framed areas in **(A)** are shown at a high magnification **(C,D)**. The colors in **(B)** represent normal tubules (green), abnormal tubules with spermatocytes (yellow), and abnormal tubules without spermatocytes (red). The roman numerals in **(B–D)** represent stages I–XII. Scales, 1 mm **(A)**, 100 μm **(C,D)**.

As described above, the normal (green) segments remained in markedly damaged seminiferous tubules of IP-30 without a significant reduction in length or number from those in IP-20. We then examined which stages of spermatogenesis are preferentially involved in these remaining normal segments. A total of 87 normal segments remaining in the three testes in IP-30 were analyzed for their cell associations to determine the stage (Figure 7). The average length of the normal segments was 2.9 ± 1.2 mm, with the longest segment being 6.45 mm and the shortest being 0.70 mm. The average number of stages involved in one segment was 4.1 ± 2.4, with the maximum number being 11 and the minimum being 1. There was no correlation between the segment length and the number of stages involved in the segment (*r* = 0.19) (data not shown). The observed proportions of individual stages were compared with those expected, which were deduced from the duration of these stages in the total cycle of mouse spermatogenesis (35 days) reported previously (Oakberg, 1956b). The proportions of stage I and XII were higher, and the proportion of stage VI was lower than the corresponding expected proportions (Table 2). In addition, the average length of the individual segment areas in stage I areas were 1.1 ± 0.9 mm, which was longer than that in any other stages (data not shown).

**TABLE 2:** Proportion of stages in normal segments in IP-30.

Stage	Mouse			Average (%)	Expectation* (%)
	#10 (%)	#11 (%)	#12 (%)		
I	39	26	15	27 ± 10	11
II–III	12	13	8	11 ± 3	13
IV	7	12	5	8 ± 3	9
V	7	1	1	3 ± 3	5
VI	1	2	0	1 ± 1	9
VII	4	8	8	7 ± 2	10
VIII	14	8	19	14 ± 5	10
IX	16	8	8	11 ± 4	7
X	8	2	9	6 ± 3	5
XI	4	2	10	5 ± 3	10
XII	14	18	18	16 ± 2	10

*Relative duration of each stage to that of a cycle in normal mouse spermatogenesis. Values are taken from Oakberg (1956b).

### Localization of the Sites of Spermatogenesis Disorder

**FIGURE 8:**
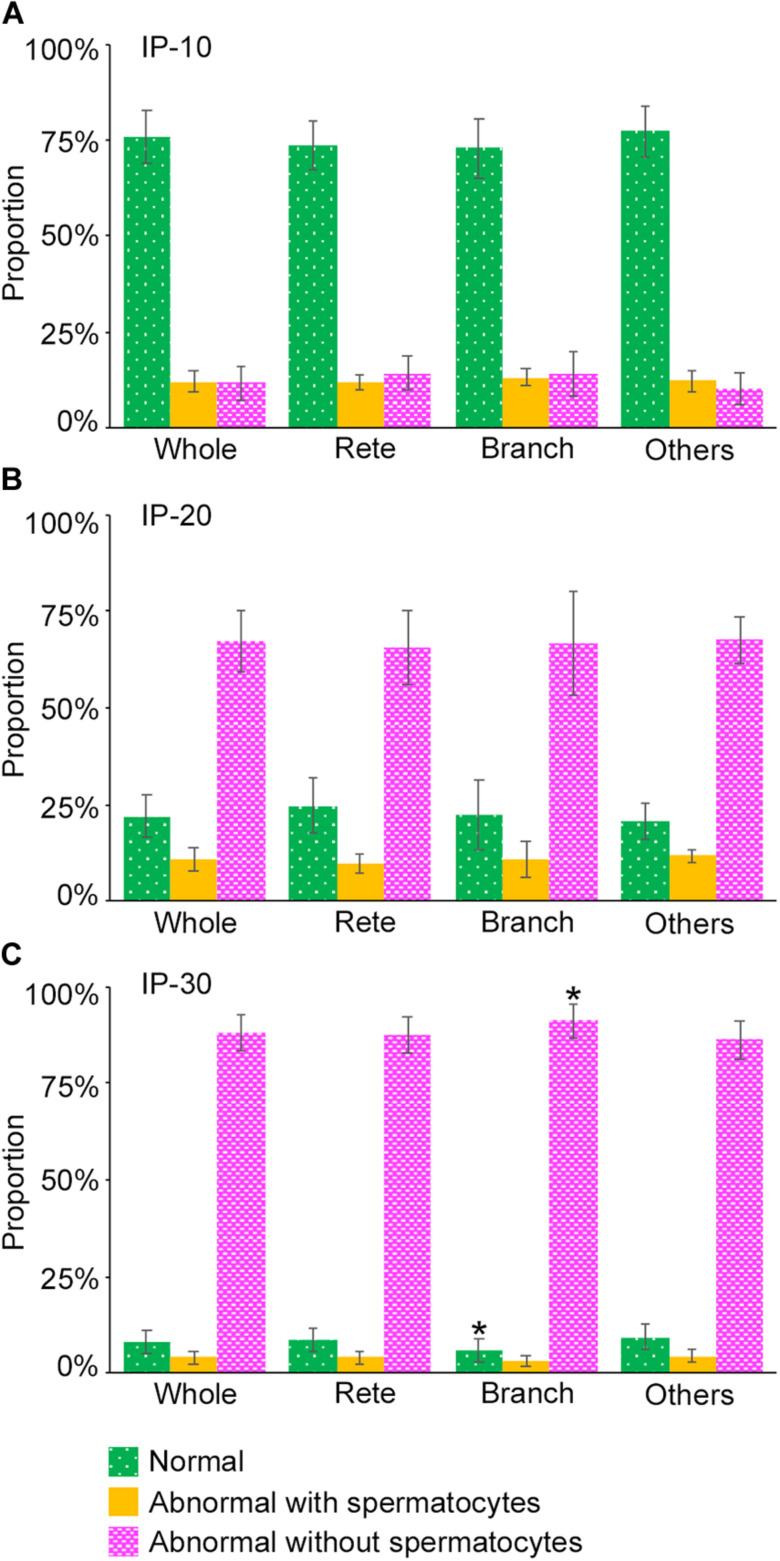
Proportions of different degrees of spermatogenesis disorder at different 3D positions in the seminiferous tubules in mice treated with different busulfan doses, IP-10 **(A)**, −20 **(B)**, and −30 **(C)**. The colors represent normal tubules (green), abnormal tubules with spermatocytes (yellow), and abnormal tubules without spermatocytes (red). The abbreviations of the positions in seminiferous tubules are as follows: whole for all tubule areas, Rete for areas within 10 mm from the terminating points, Branch for areas within 10 mm from the branching points, and Others for areas more than 10 mm apart from the terminating points and branching points. Each value represents the mean ± SD of three samples. Statistical analysis was performed between the values of Whole and those of Rete, Branch, or Others for the same colors. *Significantly different from Whole (*P* < 0.05).

Next, we examined if the degrees of spermatogenesis disorder differ among different 3D positions in seminiferous tubules. The proportions of the three color segments were measured in three different areas of seminiferous tubules, i.e., Rete (positions within 10 mm from the terminating points with the rete testis), Branch (positions within 10 mm from the branching points), and Others (positions more than 10 mm apart from the terminating points and branching points), and compared with those in the whole seminiferous tubules (Whole) (Figure 8). In IP-10 and IP-20, none of the proportions of the three colors in Rete, Branch, and Others were significantly different from those in Whole. On the other hand, in IP-30, the proportion of red segments was slightly but significantly higher in Branch than in Whole, and the proportion of green segments was slightly but significantly lower in Branch than in Whole.

**FIGURE 9:**
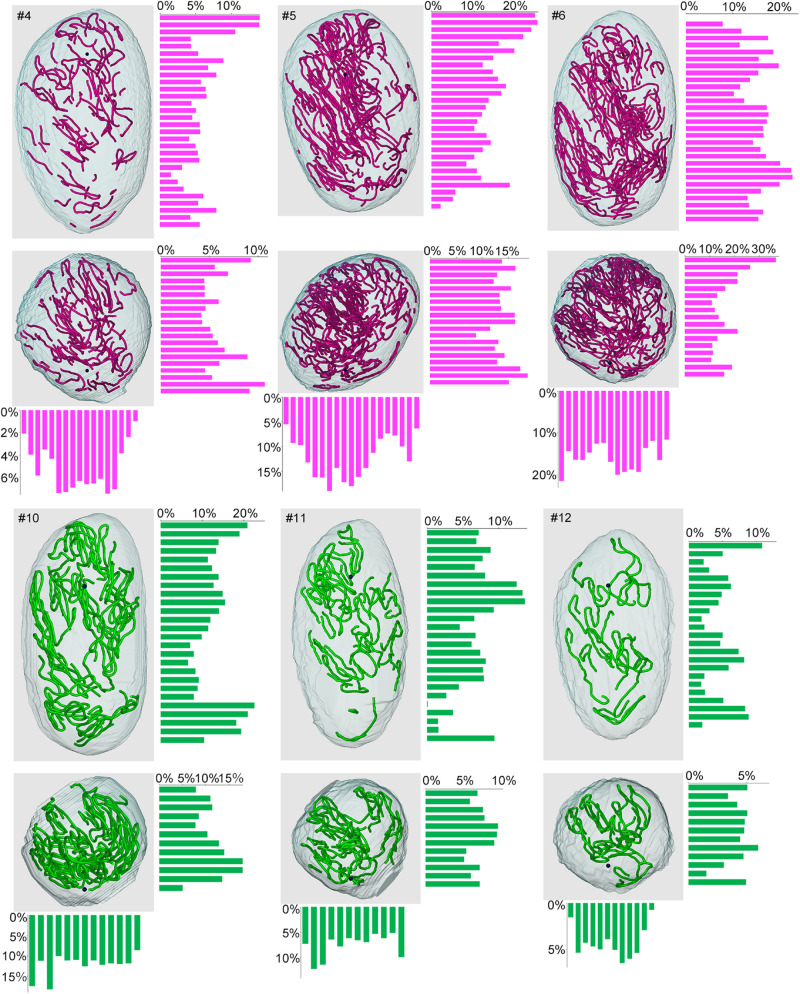
Proportions of different degrees of spermatogenesis disorder at different 3D positions in the testes from mice treated with different busulfan doses. The relative lengths of abnormal segments without spermatocytes (red) in IP-10 (#4, 5, 6) and those of normal segments (green) in IP-30 (#10, 11, 12) were measured in all tubule areas involved in 200-μm-thick serial slices of each testis parallel to the three planes formed by the *X*, *Y*, and *Z*-axes. The 3D core lines of all red or green segments in each testis, and the bars representing the proportions (%) of segments with respective colors in each slice are shown.

Lastly, we examined if the degree of spermatogenesis disorder differs among different 3D positions in a testis without considering the course of seminiferous tubules. Using the 3D reconstruction software, each testis was divided into 200-μm-thick serial slices along the perpendicular planes formed by the *X-Y-Z*-axes, and the proportions of green, yellow, and red segments in all seminiferous tubule areas involved in each slice were measured. In the present study, special attention was paid to the distribution of red (abnormal without spermatocytes) segments in IP-10 and green (normal) segments in IP-30 (Figure 9). There was a large difference in the proportion of red and green segments among individual slices in all directions. However, on comparison of three testes from three different mice belonging to the same IP-10 or IP-30 group, the positions in the slices with higher or lower proportions of red or green segments, respectively, were distributed randomly and not fixed among different testes.

## Discussion

In the present study, we reconstructed seminiferous tubules in 12 mice treated with varying doses of busulfan and analyzed the 3D distribution of spermatogenesis, in order to examine if spermatogenesis disorder occurs preferentially at specific sites in seminiferous tubules. The testis volume and total length of seminiferous tubules were reduced depending on the dose of busulfan. The positive correlation between the tubule length and the proportion of markedly impaired spermatogenesis with the highest dose of busulfan suggests that the length of seminiferous tubules is shortened due to massive degeneration. However, busulfan treatment caused no change in the basic structure of seminiferous tubules, including the numbers of tubules, terminating points, branching points, and blind ends.

Although the proportion of markedly impaired spermatogenesis increased and that of normal spermatogenesis decreased in a busulfan dose-dependent manner in all seminiferous tubules, the increased proportion of markedly impaired spermatogenesis with the highest dose of busulfan was accompanied by a decreased proportion of moderately impaired spermatogenesis and unchanged proportion of normal spermatogenesis when compared with a moderate dose of busulfan. Furthermore, analysis of the length and number of segments revealed that when the dose of busulfan increased from moderate to the highest, the segments with markedly impaired spermatogenesis increased in length, but not in number, those with moderately impaired spermatogenesis decreased in number, but not in length, and those with normal spermatogenesis remained unchanged in length and only slightly decreased in number. These phenomena suggest that the markdly damaged segments fuse with each other, whereas the moderately damaged segments further impair spermatogenesis and fuse with neighboring markedly damaged segments, resulting in an increased length of segments with markedly impaired spermatogenesis at the highest dose of busulfan. Furthermore, weakly damaged segments do not further impair spermatogenesis and instead recover and remain as segments of a certain length and number with normal spermatogenesis even at the highest dose of busulfan.

Busulfan is an alkylating agent that kills differentiated spermatogonia and spermatogonial stem cells (de Rooij and Kramer, 1970; Bucci and Meistrich, 1987). Wang et al. (2010) reported normal spermatogenesis in 93% of the seminiferous tubules in mice treated with a low dose of busulfan (10 mg/kg), but it was scarcely found with higher doses of busulfan (20 or 30 mg/kg) at 4 weeks after treatment. In the present study, approximately 76, 22, and 8% of tubule segments exhibited normal spermatogenesis at 12 weeks after treatment with 10, 20, and 30 mg/kg of busulfan, respectively. This suggested that these normal segments result from the expansion of remaining spermatogenesis or recovery of spermatogenesis from surviving stem cells that proceeded between 4 and 12 weeks. The present study also revealed that the normal segments with the highest dose of busulfan preferentially involve stages I and XII, suggesting that the seminiferous epithelia recovered from the initial damage and spermatogenesis proceeded to these stages by 12 weeks after treatment.

Regarding the 3D localization of the sites of spermatogenesis disorder, the proportion of markedly impaired spermatogenesis, although not notably different among individual ordered seminiferous tubules in a testis, was slightly but significantly higher in the tubule areas near the branching points, suggesting that these positions in the seminiferous tubules are more vulnerable to busulfan-induced damage. On the other hand, analysis of the whole testis without considering the course of seminiferous tubules revealed that both the sites of markedly impaired spermatogenesis with the lowest dose of busulfan and those of normal spermatogenesis with the maximum dose of busulfan largely differed among different positions in a testis. These sites are considered to represent the most vulnerable portions of the testis where degeneration of tubules preferentially occurs and the least vulnerable portions of testis where regeneration of tubules preferentially occurs, respectively. However, these sites were not consistent in location among different testes from individual mice.

The factors responsible for the site-specific disorder of spermatogenesis may reside outside of seminiferous tubules. In the human testis with NOA, several groups reported that the least vulnerable positions, i.e., the sperm containing-regions, receive more abundant blood supply (Har-Toov et al., 2004; Herwig et al., 2004). In the mouse testis, seminiferous tubules are uniformly surrounded by capillaries (Suzuki, 1982), thus it is unlikely that the density of capillaries influences the damage of tubules. However, the higher permeability of local capillaries may increase or ameliorate the damage by increasing the testicular concentration of busulfan or serum factors that promote recovery of tubules, respectively. Our present study suggested that the tubule areas near the rete testis, from which blood vessels enter the testis and around which tissue perfusion is considered most abundant, do not have higher or lower proportions of impaired or normal spermatogenesis. This result is consistent with the previous study by Schwarzer et al. (2013), which reported no relationship between biopsy sites near the main testicular vessels or rete testis and successful sperm retrieval in 220 cases of NOA. In addition to the factors from blood vessels, those from nerve terminals and cells surrounding seminiferous tubules, such as Leydig cells and macrophages, may be responsible for the regional difference in the degree of spermatogenesis disorder in the testis.

The present study revealed the detailed 3D structure of seminiferous tubules in busulfan-induced experimental disorder of spermatogenesis in mice. The present methods can be readily applicable to investigating other types of mouse spermatogenesis disorder, including that due to aging, which may have different mechanisms from those in the busulfan model. Although there are large limitations for comparing the mouse models with human NOA that has unknown mechanisms, our ultimate goal is application of the present methods to the study of human NOA. The structure of the testis differs between humans and mice in several aspects: the human testis, which is approximately 150-times larger in volume than the mouse testis, is divided into many lobules by connective tissue septa that separate individual seminiferous tubules, whereas no such septa exist in the mouse testis; human seminiferous tubules are considered to be composed of numerous anastomosed thin loops and run in random directions (Johnson, 1934), whereas mouse seminiferous tubules have limited anastomosis and run in zig-zag curves locally but concentrically as a whole along the circumference of the testis (Nakata et al., 2015a, 2017a). It will be of interest to examine if the degree of spermatogenesis disorder differs from lobule to lobule in human NOA, and if the tubule areas with impaired or normal spermatogenesis are preferentially located near the branching points, which are expected to exist in higher frequencies than in mice. However, obtaining the whole testis from NOA patients is difficult. Therefore, our group is currently conducting studies using human testis specimens obtained through surgery to examine the detailed and precise 3D structure of normal human seminiferous tubules. We are also planning to examine the 3D distribution of gene products inside or outside of seminiferous tubules that may control the initiation and/or expansion of spermatogenesis using normal and disordered mice of different ages. These approaches will help to elucidate the mechanism of NOA and effective clinical treatments for NOA.

## Data Availability Statement

The original contributions presented in the study are included in the article/supplementary material, further inquiries can be directed to the corresponding author/s.

## Ethics Statement

The animal study was reviewed and approved by the Kanazawa University.

## Author Contributions

HN designed the project and performed the study. HN, TN, and SI analyzed the data. HN and SI wrote the manuscript. All authors discussed the data and commented on the manuscript.

## Conflict of Interest

The authors declare that the research was conducted in the absence of any commercial or financial relationships that could be construed as a potential conflict of interest.

## Footnotes

1 http://imagej.nih.gov/ij/
